# Variations of Trail Runner’s Fitness Measures across a Season and Relationships with Workload

**DOI:** 10.3390/healthcare9030318

**Published:** 2021-03-12

**Authors:** Sérgio Matos, Filipe Manuel Clemente, Rui Silva, Joel Pereira, Pedro Bezerra, José María Cancela Carral

**Affiliations:** 1 Faculty of Educational Sciences and Sports Sciences, University of Vigo, 36005 Pontevedra, Spain; chemacc@uvigo.es; 2 Escola Superior Desporto e Lazer, Instituto Politécnico de Viana do Castelo, Rua Escola Industrial e Comercial de Nun’Álvares, 4900-347 Viana do Castelo, Portugal; filipe.clemente5@gmail.com (F.M.C.); rui.s@ipvc.pt (R.S.); joelpereira@esdl.ipvc.pt (J.P.); pbezerra@esdl.ipvc.pt (P.B.); 3 Douro Higher Institute of Educational Sciences, 4560-708 Penafiel, Portugal; 4 Instituto de Telecomunicações, Delegação da Covilhã, 1049-001 Lisbon, Portugal; 5 Unidade de Investigação e Treino em Trabalhos em Alturas e Atividades de Ar Livre, Escola Superior Desporto e Lazer, 4960-320 Melgaço, Portugal; 6 The Research Centre in Sports Sciences, Health Sciences and Human Development, 5001-801 Vila Real, Portugal

**Keywords:** endurance sports, trail running, fitness levels, performance, training monitoring

## Abstract

Trail running involves off-road running over different surfaces of positive and negative unevenness. Given these particularities and the associated physical demands, it is essential to understand this relationship and how fitness levels influence performance. This study aimed to analyze fitness level variations during different times of the season and establish a relationship between changes in fitness levels and accumulated load. Twenty-five trail running athletes (age: 36.23 ± 8.30 years) were monitored over 52 weeks. Three periods of assessment were implemented, while load between those periods was calculated. Athletes were monitored daily by global positioning systems. The collected data included distance covered, duration, and rate of perceived exertion (RPE), which were used to obtain session-RPE. Additionally, maximal aerobic speed, vertical jump, and dynamic balance were tested periodically. Moderate inverse correlations were found between assessment 1 and 2 for total sRPE and vertical jump: countermovement jump (VJ: CMJ) (*r* = −0.349), and Y balance test: left posterolateral (YBT: LPL) (*r* = −0.494). Similar correlations were found between assessment 2 and 3 for total sRPE and VJ: CMJ (*r* = −0.397), and vertical jump: drop jump (VJ: DJ) (*r* = −0.395). The results suggest that trail running coaches should monitor and assess dose–response relationships and possible anterior asymmetries of dynamic balance performance.

## 1. Introduction

Athletes of any sport aspire to achieve their best performance at the point of competition through training [1]. Although the training process is crucial, several conditions can influence an athlete’s physiological, psychological, and biomechanical factors—even their genetics and age [1]. The dose–response effects resulting from training’s relationship with all associated factors can either enhance or hinder performance [2]. The complexity of the relationship between training and an athlete’s physical and physiological levels is considered crucial throughout the training process [3]. In this sense, monitoring the training load is essential for determining the dose–response relationship between training and the athlete [4]. Thus, such monitoring can reveal the ideal balance that will improve the athlete’s performance [5].

Training load can be monitored based on either internal or external load [6]. External load is related to the work done by the athlete (e.g., distance, acceleration, and speed), while internal load has to do with the athlete’s biological responses to the workload (e.g., heart rate, subjective perceived exertion) [6]. Monitoring training load makes it possible to verify whether specific training stimuli provide the appropriate variations in fitness levels and allow coaches to make specific adjustments for any individual athlete [7]. Such adjustments must be made based on the period of the season if they are to help the athlete to adapt to the stimuli optimally and achieve top physical condition at the time of competition [8].

The best way to adjust stimuli varies from one athlete to the next; therefore, athletes’ responses to loads accumulated over the season must be accurately monitored by the team, coach, or athlete using reliable measures, such as the subjective perception of effort [9]. Rate of perceived exertion (RPE), a concept developed by Borg [10], is a valid and reliable measure of the load to which athletes are exposed, and it has been used in several sports [7,11,12,13,14]. The session-RPE (sRPE) derives from the value from the RPE and the duration of the training session (in minutes) and is calculated as the formula: value of RPE multiplied by the duration of the training session (in minutes) [15]. Thus, regardless of the sport, it is crucial to understand athletes’ training methods and the relationships between fitness levels, training, and competition performance [16].

Trail running is performed on uneven off-road routes (e.g., through mountains, forests, deserts), depending on the distance or elevation of any given race [17]. Given these particularities, the athlete’s demands are higher due to the number of kilometers, uneven ground, and constant ascents and descents; it is essential to understand this relationship and which fitness levels most strongly affect performance [18]. At the same time, it is essential to understand what physical variables (e.g., strength and balance) should be included in the training process to enhance the athlete’s performance. The decrease in muscle strength in sports such as running is notorious, suggesting that improvements in eccentric muscle strength may be decisive in performance [19]. The relationship between strength training and endurance sports performance has shown that adequate strength levels can improve the running economy [20,21]. The same study revealed that 1–2 strength training sessions per week will improve the running economy and the athlete’s performance [21]. If, on the one hand, it is important to incorporate strength training into the training process, it seems imperative to understand the effects on the athlete over time, with some studies developed over five months with weightlifters [22], with skiers for eight weeks [23] and with footballers for six weeks [24]. For strength assessment, vertical jumps such as countermovement jump and drop jump are valid and reliable tests performed with force platforms or contact mats [25].

Additionally derived from running as a predominantly dynamic activity, balance is preponderant in postural control [26], essential in trail running due to uneven surfaces requiring the athlete to constantly correct posture to compensate for the imbalance [27]. In addition to running, some balance investigations have been carried out in other sports such as football [24], skiing [23], volleyball [28], and weight lifting [22]. In this sense, there is evidence that balance training provides increases in muscle power, which is crucial for motor skills performance [29]. Due to their validity and reliability, some instruments as the Balance Error Scoring System and the Y Balance Test are used in the literature as instruments for assessing balance [30,31].

However, regarding the trail running demands, Lazzer et al. (2012) analyzed the effects on running energy cost and VO2max consumption during an ultra-endurance competition and how they can determine the performance [32]. A study conducted over four weeks with competitions [11] revealed moderate and negative correlation coefficients (*r* = −0.395, *p* = 0.016) between VO2max and pace. Corroborating this evidence, another study on trail running [33] observed a negative correlation between VO2max and race time (*r* = −0.78, *p* = 0.02), suggesting that adequate cardiorespiratory fitness levels translate to improvements in pace and, consequently, performance.

However, other variables were revealed to be predictors of performance, such as maximal aerobic speed (MAS), the fraction of maximal aerobic speed (FMAS), and knee extensor force, which explained a combined 98% of performance variations in a study conducted on ultra-mountain endurance event runners [34]. In another study [11], the only differences found regarding loads between the four weeks were for the weekly average RPE (*p* = 0.011; η^2^ = 0.078, small effect) and sRPE (*p* = 0.025; η^2^ = 0.065, small effect), suggesting a lack of stimulation in the athlete, which is essential to optimize performance [35].

Although variations in workload indices are necessary to understand performance, the literature show that between-week changes in workload of more than 10% are related to injury risk and can affect the performance [36]. In this sense, and assessing the fitness levels, Matos et al. (2020) demonstrated that the three weeks before an injury occurred were marked by significant weekly increases in workload indexes (acute load; acute: chronic workload ratio; training monotony; and training strain for sRPE, total distance, and training time) [37].

Such evidence reveals the importance of adequate fitness levels for optimizing performance and suggests that what is considered optimal might differ at different moments of the season. In trail running, a sport with several competitive moments, accumulated load should be considered throughout the season.

However, the relationship between variations in fitness levels and accumulated load is not well-established. Therefore, this study aimed to: (i) analyze variations in fitness levels at different points of the season; and (ii) establish a relationship between changes in fitness level and accumulated load.

## 2. Materials and Methods

### 2.1. Participants

The study included 25 male trail running athletes (age: 36.23 ± 8.30 years old; height: 172.12 ± 5.12 cm; body mass: 67.24 ± 5.97 kg), with a minimum of 600 International Trail Running Association (ITRA) ranking points who had participated in trail running championships in Portugal in the 2018/2019 season.

Over 52 weeks (a trail running season), all athletes reported data related to training sessions as defined by the athletes or coaches themselves. All athletes were monitored daily using global positioning systems (GPSs), which collected data for distances covered and duration. Additionally, RPE was measured and used to obtain session-RPE.

The inclusion criteria necessary for participation in the study were: (i) participation in the Portuguese national trail running championships; (ii) more than three years of experience in the sport; (iii) registration in all training sessions; and (iv) not being injured for more than three consecutive weeks in the last 12 months. All athletes had prior knowledge of and were informed about the study’s objectives, procedures, and protocols. They all signed an informed consent form freely and voluntarily. The study followed the Helsinki Declaration’s ethical recommendations for studies in humans.

### 2.2. Experimental Approach to the Problem

**Figure 1:**
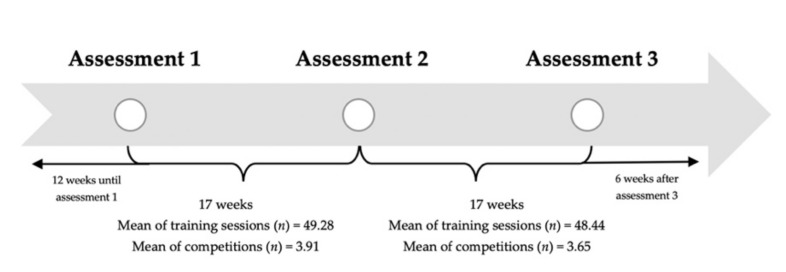
Timeline of assessments during 52 weeks of study.

This research followed a cohort study design. Over the 52 week study period, 148.12 ± 57.53 training sessions for each athlete were monitored and analyzed. The athletes or their coaches determined the nature of training sessions. Monday was considered the first day of each week, and Sunday was considered the last day. Anthropometric, vertical jumps, dynamic balance and aerobic performance assessments were performed every four months (Figure 1).

The assessments were carried out in the morning, always in the same place and in the following order after a five-minute warm-up: (i) anthropometrics; (ii) Y balance test (YBT); (iii) countermovement jump (CMJ); (iv) drop jump (DJ); (v) aerobic performance. The evaluations took place 72 h after the last training session or competition. Evaluations of the body composition, Y balance test, CMJ, and DJ were performed in a 10 m^2^ room, and a track was used for aerobic performance evaluations. During all assessments, there was a temperature of 16 °C, a relative humidity of 45%, and no precipitation.

### 2.3. Periodic Assessment

#### 2.3.1. Anthropometrics

Tanita BC-601 (measured to the nearest 0.1 kg, Tokyo, Japan) was used to obtain body mass, and a stadiometer (measured to the nearest 0.1 cm, Seca 217, Hamburg, Germany) was used to measure height.

#### 2.3.2. Vertical Jump Assessment

CMJ was used to assess the lower limbs’ explosive strength, and DJ was used to evaluate reactive jumping ability. This test is a gold standard to measure the vertical height jump with particular emphasis on the stretching–shortening cycle. The evaluations were performed using a DIN-A2 contact platform (Chronojump, Spain) connected to a Chronopic 3 microcontroller and a computer that allowed the analysis of the results obtained through the appropriate software. Considering the frequencies at which the microcontroller was tested, the average absolute errors were 0.13% for contact time and 0.14% for flight time. The difference between the DIN-A2 contact platform and the Ergojump-Boscosystem platform was only 1.40 ± 0.92%, and the intraclass correlation coefficient (ICC) was 0.95.

The CMJ test was performed with the athletes standing on a platform with both hands on their hips, which allowed them to assume a comfortable depth of knee flexion during the descending phase. During the jump’s flight phase, the athletes maintained hip and knee extensions and jumped as high as possible without removing their hands from their hips. They had to land in the same place with both feet simultaneously.

The DJ test was performed with athletes starting on a 30 cm-tall box, keeping their hands on their hips. The athletes were instructed to start the test by moving one foot off the box and then moving their whole body in a downward direction towards the contact platform. They were then to perform a touch movement on the platform, which enabled upward movement (jump) while maintaining hip and knee extension. In the second moment of contact with the platform, athletes had to land with both feet simultaneously and assume a half-squat position to minimize the jump’s impact.

The athletes performed three jumps for both tests. All results were recorded, but only the highest jump value was considered for analysis. A jump was repeated if the athlete moved their hands from their hips or flexed their hips or knees during the jump. The outcomes related with these tests were: (i) CMJ height (cm); and (ii) DJ height (cm).

### 2.4. Dynamic Balance

The dynamic postural balance test was performed using the same device used for the Y balance test (Move2Perform, Evansville, IN, USA). The test was selected based on the validity and reliability to measure the dynamic balance. Following the protocol described by Plisky et al. [38], the athletes performed the test with one foot on the central plate while they reached with the free foot along the anterior, posteromedial, and posterolateral axes.

The test was attempted three times for each foot, starting with the right foot being the foot on the central plate. The best result for each foot and each axis was collected and recorded as the best attempt. The athlete was instructed to repeat a trial if: (i) they lost their position by touching the ground or another surface; (ii) their free foot was placed over the movable plate; (iii) they did not keep the foot in contact with the moving plate until the end of the movement; or (iv) they failed to return to the initial position in a controlled manner after reaching all axes. The outcome related with this test was YBT length (cm).

### 2.5. Aerobic Performance

According to the protocol described by Berthon et al., athletes participated in a five-minute field test that is reliable for assessing aerobic performance [39]. The warm-up consisted of five minutes of running at a low and comfortable pace. The test was run on a track during the morning, with temperatures ranging from 15–25 °C, depending on the day and time. During the test, athletes were advised to maintain a steady pace to reach maximum performance, which eliminated moments of rest from the test. The total distance covered was recorded, and the maximal aerobic speed (MAS) (expressed in m/s) was calculated by dividing the total distance (in meters) by time (in seconds). Thus, the final outcome was MAS meters per second (m/s). The field-based test was used because of the validity of obtaining the MAS of the athletes in their specific conditions (outdoor running).

### 2.6. Training Load Monitoring

#### 2.6.1. Distance Covered

The distances covered by the athletes during the training sessions were recorded using watches with GPS technology (Polar V800 watch—37 mm × 56 mm × 12.7 mm, weight: 79 g, Polar, Finland). Previous studies have shown that the Polar V800 watch has acceptable accuracy values, which was the main reason for its choice in the present study [40]. The outcome related with the data collection was the distance in kilometers (km).

#### 2.6.2. Rate of Perceived Exertion

Rate of perceived exertion was recorded during all training sessions through a procedure performed by athletes 30 min after each training session based on their responses to the question, “How hard was the training session?” The Borg CR-10 scale [10] was used to record and determine each athlete’s response; this scale was introduced to the athletes two weeks before the study to ensure their familiarity with it and ability to provide precise answers. For all training sessions, the values of RPE and the duration of the training session (in minutes) were used to calculate session-RPE (expressed in arbitrary units (A.U.)) [15], which is a metric that quantifies internal load [41]. The outcome related with the data collection was sRPE arbitrary units (A.U.).

### 2.7. Statistical Analysis

Results (given as means and standard deviations) are expressed as text, as well as in tables and figures. All data were checked for normality and homogeneity (for *p* > 0.05), and a repeated-measures ANOVA was subsequently performed to compare the fitness levels and training load variables between assessments. Bonferroni’s post hoc test was used to analyze pairwise variations (assessment vs. assessment analysis). The level of statistical significance was set at *p* < 0.05.

Additionally, the standardized effect size (ES) of Cohen’s *d* was calculated for pairwise comparisons. The Pearson correlation test (*r*) was used to assess the associations between training load variables and fitness variables (based on the mean values of each assessment). The magnitudes of the correlation were categorized based on the following thresholds: <0.1 (trivial), from 0.1 to 0.3 (small), from 0.3 to 0.5 (moderate), from 0.5 to 0.7 (large), from 0.7 to 0.9 (very large), and ≥0.9 (nearly perfect) [42]. SPSS Statistics software (version 24, IBM Corporation, Armonk, NY, USA) was used for the analysis.

## 3. Results

The repeated measures ANOVA tested the variation of fitness variables across the three assessment periods; the results revealed significant differences between the first and third assessments for the variables YBT: LPM (*p* < 0.001), YBT: RPL (*p* = 0.005), and YBT: LPL (*p* < 0.001), and also between the second and third evaluation moments for the YBT: LPL (*p* = 0.002). Descriptive statistics and pairwise comparisons (between assessment periods) can be found in Table 1.

Descriptive statistics of accumulated training loads between periods of assessment can be found in Table 2. No significant differences were found between periods of assessment for the different workload measures (*p* > 0.05).

**Figure 2:**
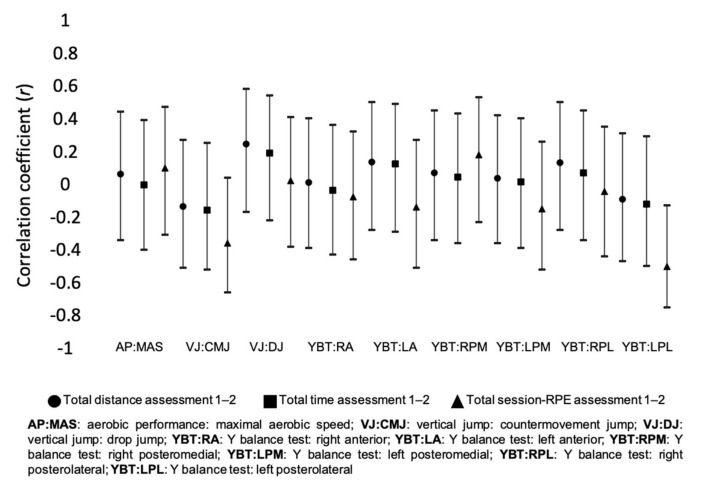
Correlation of accumulated training load variables between assessment 1 and 2.

Figure 2 presents the correlation coefficients between training load measures and the percentage of changes of different fitness variables between the assessment periods 1 and 2. Moderate inverse correlations were found between total sRPE and VJ: CMJ (*r* = −0.349; *p* = 0.102), and YBT: LPL (*r* = −0.494; *p* = 0.020). Small inverse correlations were found between total distance and VJ: CMJ (*r* = −0.125; *p* = 0.553), and small positive correlations were found in VJ: DJ (*r* = 0.252; *p* = 0.224), YBT: LA (*r* = 0.143; *p* = 0.495) and YBT: RPL (*r* = 0.141; *p* = 0.500). Small inverse correlations were found between total time and VJ: CMJ (*r* = −0.148; *p* = 0.480), and YBT: LPL (*r* = −0.113; *p* = 0.599), and small positive correlations were found in VJ: DJ (*r* = 0.199; *p* = 0.341), and YBT: LA (*r* = 0.134; *p* = 0.523). Small inverse correlations were found between total sRPE and YBT: LA (*r* = −0.129; *p* = 0.559), and YBT: LPM (*r* = −0.141; *p* = 0.522), and small positive correlation were found in AP: MAS (*r* = 0.106; *p* = 0.640), and YBT: RPM (*r* = 0.189; *p* = 0.388).

**Figure 3:**
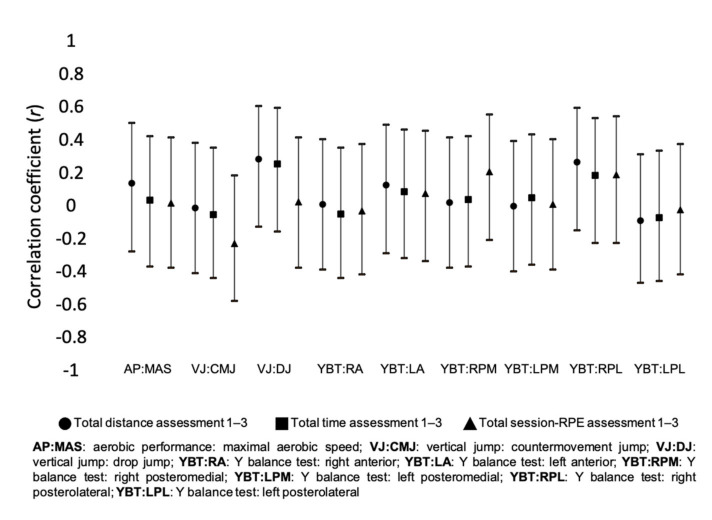
Correlation of accumulated training load variables between assessment 1 and 3.

Figure 3 presents the correlation coefficients between training load measures and the percentage of changes of different fitness variables between the assessment periods 1 and 3. Small positive correlations were found between total distance and AP: MAS (*r* = 0.144; *p* = 0.493), VJ: DJ (*r* = 0.290; *p* = 0.168), YBT: LA (*r* = 0.131; *p* = 0.533), and YBT: RPL (*r* = 0.273; *p* = 0.186). Small positive correlations were found between total time and VJ: DJ (*r* = 0.262; *p* = 0.216), and YBT: RPL (*r* = 0.191; *p* = 0.360). Small inverse correlations were found between total sRPE and VJ: CMJ (*r* = −0.223; *p* = 0.305), and small positive correlations were found in YBT: RPM (*r* = 0.212; *p* = 0.320), and YBT: RPL (*r* = 0.196; *p* = 0.359).

**Figure 4:**
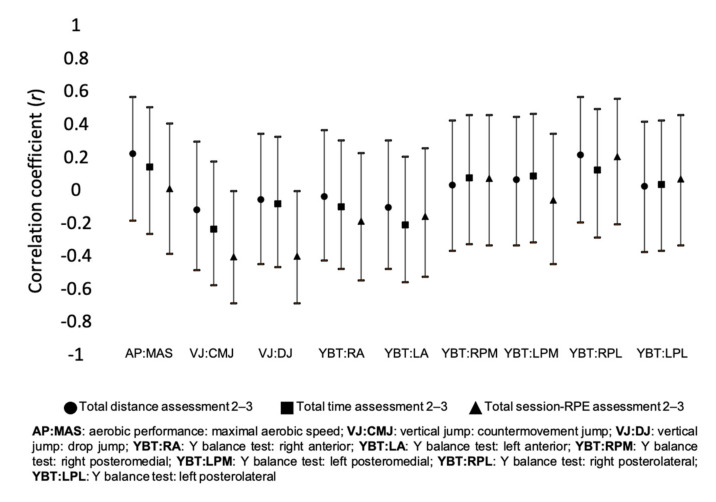
Correlation of accumulated training load variables between assessment 2 and 3.

Figure 4 presents the correlation coefficients between training load measures and the percentage of changes of different fitness variables between the assessment periods 2 and 3. Moderate inverse correlations were found between total sRPE and VJ: CMJ (*r* = −0.397; *p* = 0.049), and VJ: DJ (*r* = −0.395; *p* = 0.051). Small inverse correlations were found between total distance and VJ: CMJ (*r* = −0.111; *p* = 0.596), and small positive correlations were found in AP: MAS (*r* = 0.229; *p* = 0.281), and YBT: RPL (*r* = 0.219; *p* = 0.292). Small inverse correlations were found between total time and VJ: CMJ (*r* = −0.228; *p* = 0.273), and YBT: LA (*r* = −0.204; *p* = 0.327), and small positive correlations were found in AP: MAS (*r* = 0.148; *p* = 0.490). Small inverse correlations were found between total sRPE and YBT: RA (*r* = −0.182; *p* = 0.383), and YBT: LA (*r* = −0.154; *p* = 0.463), and small positive correlations were found in YBT: RPL (*r* = 0.208; *p* = 0.317).

## 4. Discussion

**Figure 5:**
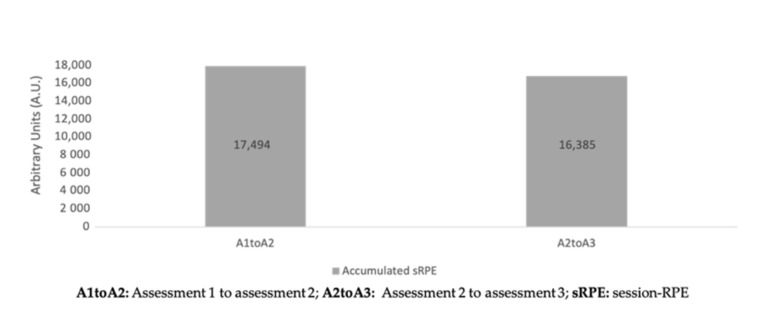
Accumulated sRPE during the periods of the season.

**Figure 6:**
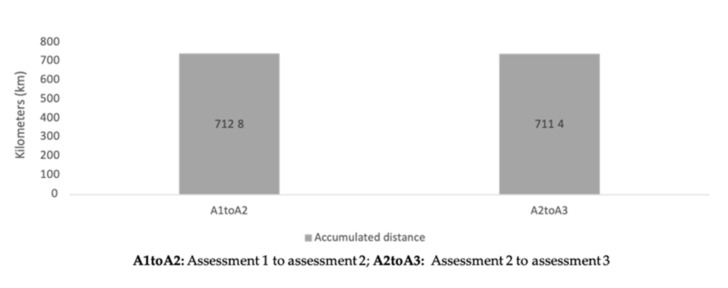
Accumulated distance during the periods of the season.

**Figure 7:**
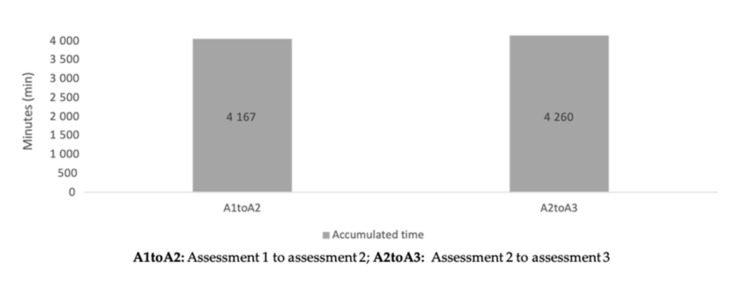
Accumulated time during the periods of the season.

The aims of the present study were to analyze the variations of fitness levels at different moments of the trail running season and to establish a relationship between those variations and the accumulated load throughout the season. The main finding was that no significant differences were found between assessments for training load (Figure 5, Figure 6 and Figure 7) and fitness variables, except in terms of dynamic balance measures. Moderate inverse correlations were found between sRPE and vertical jumps, from A1 to A2 and from A2 to A3 assessments.

Regarding fitness level variations, no significant changes occurred between the different moments of the season for aerobic and jump performances. Additionally, in a study with elite runners during a season, no significant differences were found for force production, possibly due to the high volume of resistance training characteristics of long-distance athletes, which may influence strength gains [43]. On the other hand, the posterior movements (medial and lateral) of both lower limbs during the dynamic balance performance test revealed significant positive changes over the 52-week training period. These results are similar to another study, but with skiers, where significant improvements were found in all directions in the YBT for the experimental group (with neuromuscular training), while in the control group, the results significantly worsened [23]. Additionally, there was a lack of meaningful changes for accumulated loads (sRPE, total distance, and total time) between assessments.

No improvements were found in jump performance over the season, possibly because the running stimuli imposed on trail runners might not be sufficient to improve the stretch-shortening cycle, which could also be due to accumulated fatigue [44,45]. Corroborating these results, in another study, no significant differences were found in jump performance, suggesting that the lack of high-intensity training may have influenced the jump performance [23].

On the other hand, although there were no significant differences for training loads between assessments, an accumulation of loads could result in increased aerobic performance [46]. However, this was not the case in the present study. In fact, in a study conducted on 27 professional soccer players over 10 weeks of training, it was found that maximal aerobic speed did not change between pre- and post-assessments [7]. Even though that study is in concordance with the lack of changes in aerobic performance in the present study, the duration and the type of sport were different.

Furthermore, a possible explanation for the lack of change between assessments could be that changes in aerobic performance seem to occur mainly for high-intensity (>90% HRmax) actions [47]. Thus, trail running coaches and athletes should consider incorporating high-intensity sessions during the training process, because they seem to improve aerobic fitness, running economy, and muscular adaptations without increasing neuromuscular strain [47]. Additionally, strength training and plyometric exercises might help to improve jump performance, running economy, and time to exhaustion at maximal aerobic speed [48]. However, the type of high-intensity and strength sessions should be carefully planned and individualized.

Considering the second aim of the present study, the small to moderate inverse correlations between accumulated loads (sRPE, total distance, and total time) and jump performance revealed a lower percentage of change in jump performance when the accumulated loads were higher. This suggests that training with higher intensities and lower volumes may be more effective for endurance athletes’ strength gains [49]. This was more prominent when considering the internal load (sRPE) alone and jump performance; these were the only variables that showed moderate inverse correlations between A1 and A2 and between A2 and A3. This evidence was also demonstrated in a study with elite runners, where sRPE correlated significantly with force production in medium- and long-distance athletes [43].

As mentioned earlier, this lower change in jump assessments might be due to the lack of stimuli for running itself, which could make it impossible to improve jump performance. However, the moderate inverse correlation between sRPE and jumping could be caused by chronic fatigue [50]. In fact, a previous study analyzed the associations between perceived loads and physical fitness in 19 professional soccer players over nine weeks of training and competition [51]. The findings revealed large negative correlations between accumulated sRPE and jump performance, which is coincident with our results [51].

Notwithstanding, trail running is unique in that it involves an accumulation of varying elevations, accelerations, and decelerations, depending on whether a competition falls into the lower or greater distance category. Those metrics, especially the accelerometry ones, might adversely affect running mechanics, thereby leading to a greater frequency of steps and leg stiffness due to chronic fatigue and, in turn, decreasing neuromuscular performance [52,53].

Although the overall external load variables and the percentage of change in fitness variables showed only small correlations, the small positive correlations between accumulated loads of sRPE, total distance, total time, and AP: MAS are noteworthy. Indeed, Clemente et al. (2019) showed a small positive correlation between the sum of training loads (sRPE only) and AP: MAS [7], which corroborates our results.

Conversely, a study conducted on 10 athletes of a different running-based sport (rugby) examined the dose–response relationships between training load and the changes in aerobic performance [54]. In that work, a large negative relationship was found between high-speed running and changes in aerobic performance, suggesting that greater time spent at higher-intensity distances decreases AP: MAS [54]. That study [54], however, also showed ambiguous correlations between total distance and AP: MAS, as was also evidenced in the present study.

Furthermore, total time and sRPE had small negative correlations with the percentage of change of anterior movements (YBT: RA and YBT: LA) on the balance performance test. This fact also suggests that decreased anterior balance performance can occur because of the chronic fatigue derived from the accumulation of total time and sRPE. Additionally, the percentage change of posterior balance movements seems to increase slightly when external and internal loads increase. As such, it can be argued that after weeks of training and competition, knee-dominant movements that require prominent foot dorsiflexion are more strongly negatively affected than hip-dominant movements in trail running athletes. This evidence indicates that trail running is a multifactorial sport that involves a risk of non-contact injuries [37]. It also suggests that the anterior movement asymmetries on the Y balance test were previously associated with an increased risk of injury [55]. For those reasons, coaches and athletes should consider monitoring anterior asymmetries to reduce the risk of injury.

The present study is not without some limitations. Perhaps the most notable limitations are the small sample size and the exclusion of female athletes. Another limitation is that we analyzed only total distance and time metrics; it would be interesting to include accelerometry metrics to investigate their relationships with physical fitness. Additionally, future studies should analyze training load’s relationship with limb asymmetries during balance tests. Finally, time-series analysis should be applied in future research to eliminate the effect of season on performance.

## 5. Conclusions

The assessments carried out in the present study showed that fitness level variations are associated with significant changes in the posterior movements of the dynamic balance test, while aerobic and jump assessments showed no differences. External and internal accumulated load variables did not change between assessments. The dose–response relationships revealed moderate inverse correlations between sRPE and the percentage change in jump performance. Additionally, small inverse correlations were found between sRPE and anterior balance movement changes, while the percentage change of posterior movements increased as internal and external loads increased. The results suggest that trail running coaches should monitor and assess dose–response relationships and possible anterior asymmetries of dynamic balance performance.

## Figures and Tables

**Table 1:** Descriptive statistics of fitness variables assessed in the three periods of the season.

	A1 M ± SD	A2 M ± SD	A3 M ± SD	A2−A1 (%)|*p*|ES	A3−A1 (%)|*p*|ES	A3−A2 (%)|*p*|ES
AP: MAS (m/s)	4.8 ± 0.4	4.8 ± 0.4	4.9 ± 0.4	0.3|>0.999|0.000	1.3|0.531|0.250	1.2|0.554|0.250
VJ: CMJ (cm)	25.3 ± 5.5	25.9 ± 5.2	25.6 ± 4.9	3.5|0.671|0.112	2.2|>0.999|0.058	−1.0|>0.999|−0.059
VJ: DJ (cm)	21.0 ± 3.9	20.5 ± 4.1	21.3 ± 3.7	−0.2|>0.999|−0.125	4.5|>0.999|0.079	5.6|0.375|0.205
YBT: RA (cm)	59.6 ± 5.2	59.2 ± 4.8	59.6 ± 4.1	−0.4|>0.999|−0.080	0.5|>0.999|0.000	1.0|>0.999|0.090
YBT: LA (cm)	59.9 ± 6.0	59.6 ± 6.4	59.0 ± 4.5	−0.3|>0.999|−0.048	−1.0|0.990|−0.170	−0.5|>0.999|−0.109
YBT: RPM (cm)	102.4 ± 6.7	104.1 ± 4.9	105.3 ± 6.5	1.8|0.257|0.290	3.0|0.091|0.439	1.2|0.513|0.209
YBT: LPM (cm)	100.5 ± 8.6	102.9 ± 6.8	104.6 ± 7.3	2.8|0.107|0.310	4.4|<0.001|0.514	1.7|0.053|0.241
YBT: RPL (cm)	98.6 ± 7.8	100.7 ± 5.0	103.0 ± 6.5	2.5|0.237|0.321	4.8|0.005|0.613	2.3|0.058|0.397
YBT: LPL (cm)	98.9 ± 9.8	100.3 ± 7.6	104.9 ± 8.9	1.9|0.911|0.160	6.4|<0.001|0.641	4.7|0.002|0.556

M: mean; SD: standard deviation; A1: first assessment; A2: second assessment; A3: third assessment; (%): percentage of change; *p*: *p*-value; ES: standardized effect size of Cohen’s *d*; AP: MAS: aerobic performance: maximal aerobic speed; VJ: CMJ: vertical jump: countermovement jump; VJ: DJ: vertical jump: drop jump; YBT: RA: Y balance test: right anterior; YBT: LA: Y balance test: left anterior; YBT: RPM: Y balance test: right posteromedial; YBT: LPM: Y balance test: left posteromedial; YBT: RPL: Y balance test: right posterolateral; YBT: LPL: Y balance test: left posterolateral; m: meters; s: seconds; cm: centimeters.

**Table 2:** Descriptive statistics of accumulated training load variables registered in the three periods of the season.

	A1–A2 M ± SD	A2–A3 M ± SD	A1–A3 M ± SD	A2–A3 − A1–A2 (%)|*p*|ES
Accumulated sRPE (A.U.)	17,494.3 ± 14,947.8	16,385.4 ± 7856.3	33,879.7 ± 21,007.5	−15.4|0.686|−0.093
Accumulated distance (km)	712.8 ± 394.9	711.4 ± 281.8	1424.2 ± 625.7	−15.7|>0.999|−0.004
Accumulated time (min)	4166.9 ± 2233.6	4260.0 ± 1602.9	8426.9 ± 3487.1	21|>0.999|0.048

M: mean; SD: standard deviation; A1: first assessment; A2: second assessment; A3: third assessment; (%): percentage of change; *p*: *p*-value; ES: standardized effect size of Cohen’s *d*; sRPE: session-rated perceived exertion; A.U.: arbitrary units; km: kilometers; min: minutes.

## Data Availability

Not applicable.
